# Regulatory and Functional Involvement of Long Non-Coding RNAs in DNA Double-Strand Break Repair Mechanisms

**DOI:** 10.3390/cells10061506

**Published:** 2021-06-15

**Authors:** Angelos Papaspyropoulos, Nefeli Lagopati, Ioanna Mourkioti, Andriani Angelopoulou, Spyridon Kyriazis, Michalis Liontos, Vassilis Gorgoulis, Athanassios Kotsinas

**Affiliations:** 1 Molecular Carcinogenesis Group, Department of Histology and Embryology, Medical School, National Kapodistrian University of Athens (NKUA), 75 Mikras Asias Str., Goudi, GR-11527 Athens, Greece; a.papaspyropoulos@med.uoa.gr (A.P.); nlagopati@med.uoa.gr (N.L.); ioanna.mourkioti@gmail.com (I.M.); andriani.an22@hotmail.com (A.A.); spkyriaz@gmail.com (S.K.); mliontos@gmail.com (M.L.); 2 Biomedical Research Foundation, Academy of Athens, GR-11527 Athens, Greece; 3 Oncology Unit, Department of Clinical Therapeutics, Medical School, National and Kapodistrian University of Athens, Alexandra Hospital, GR-11528 Athens, Greece; 4 Molecular and Clinical Cancer Sciences, Manchester Cancer Research Centre, Manchester Academic Health Sciences Centre, University of Manchester, Manchester M20 4GJ, UK; 5 Center for New Biotechnologies and Precision Medicine, Medical School, National and Kapodistrian University of Athens, GR-11527 Athens, Greece

**Keywords:** long non-coding RNAs, double-strand breaks (DSB), DNA damage response and repair (DDRR), tumorigenesis

## Abstract

Protection of genome integrity is vital for all living organisms, particularly when DNA double-strand breaks (DSBs) occur. Eukaryotes have developed two main pathways, namely Non-Homologous End Joining (NHEJ) and Homologous Recombination (HR), to repair DSBs. While most of the current research is focused on the role of key protein players in the functional regulation of DSB repair pathways, accumulating evidence has uncovered a novel class of regulating factors termed non-coding RNAs. Non-coding RNAs have been found to hold a pivotal role in the activation of DSB repair mechanisms, thereby safeguarding genomic stability. In particular, long non-coding RNAs (lncRNAs) have begun to emerge as new players with vast therapeutic potential. This review summarizes important advances in the field of lncRNAs, including characterization of recently identified lncRNAs, and their implication in DSB repair pathways in the context of tumorigenesis.

## 1. Introduction

An intact genome is vital for the survival of living organisms as it ensures normal homeostasis [1]. To safeguard its integrity, cells have deployed complex signaling networks that permanently monitor, detect and respond to genotoxic insults that alter its structure and, particularly, the encoded biological information [1,2]. Collectively known as DNA damage response and repair (DDRR) pathways, these circuits represent an intense field of investigation, especially from the perspective of pathological conditions, as their failure results in the accumulation of genetic alterations that are disease-causing factors [1,2]. At the cellular level, DDRR is executed through several stages: (i) DNA damage sensing by sensors, (ii) signal transduction by transducer complexes, (iii) damage repair by effector molecules and (iv) effectors regulating downstream global responses [1,2].

Most of the known factors that build these regulatory circuits are identified as proteins. However, mounting evidence has revealed an unexpected new class of players to participate in genome surveillance pathways. These new factors constitute a subset of RNA molecules defined as non-coding RNAs (ncRNAs) [1,3]. NcRNAs comprise a very abundant component of the transcriptome that includes various subcategories, such as the long and short ncRNAs that differentiate based on their size. Despite not encoding for proteins, a growing body of evidence has revealed that ncRNAs hold an intricate role in many cellular functions and responses, expanding the central dogma of biology [3].

In the current review, we focus on the role that long non-coding RNAs (lncRNAs) play as versatile tools in the various DDRR pathways, and specifically in the detection and repair of DNA double-strand breaks (DSBs), the most deleterious type of DNA damage [1,4]. Principally, we explore which lncRNAs have been so far found to participate in DDRR, and at what level. Lastly, we examine how deregulation of these factors relates to human pathology, particularly cancer onset and progression.

## 2. Transcriptome and Long Non-Coding RNA

While a very large proportion of the human genome is transcribed [5,6,7], only an extremely small fraction is translated into proteins [3,8]. Most of the non-translated RNAs belong to a group defined as non-coding RNAs (ncRNAs) that was initially thought to represent a “dark matter in the genome” [3,5,9]. Currently, the ncRNAs comprise two broad groups, namely long and short ncRNAs (lncRNA and sncRNA, respectively), which are separated based on an arbitrary size division. Specifically, sncRNAs have a length of less than 200 nucleotides (nt) and constitute a collection of distinct subgroups, such as microRNAs (miRNAs), small interfering RNAs (siRNAs), Piwi-interacting RNAs (piRNAs), small nucleolar RNAs (snoRNAs) and other short RNAs playing important roles in translation regulation and gene silencing [10]. On the other hand, lncRNAs are transcripts longer than 200 nt and appear to be a highly abundant type of ncRNAs in the human transcriptome [11]. A further size-based division of lncRNAs involves small-lncRNAs (200–950 nt), medium-lncRNAs (950–4.800 nt) and large-lncRNAs (>4.800 nt) [10].

Notably, while large numbers of curated human lncRNA entries are available in public repositories, starting from 16.000 up to 270.044, only a relatively small proportion of them have been functionally annotated [11,12]. LncRNAs have been found to be involved in gene regulation at various levels, including epigenetic, transcriptional, post-transcriptional and translational regulation [11,13], as well as in other biological functions, such as DNA replication timing and chromosome stability, protein localization, cell cycle regulation, apoptosis and regulation of organelle function [3,10,11,14,15,16,17].

The expression levels of lncRNA genes exhibit high cell-to-cell variation [18], while the majority of lncRNAs are tissue-specific [19]. Moreover, lncRNAs demonstrate high developmental stage [20] and cell subtype specificity in heterogeneous tissues [21].

Most lncRNA species are transcribed by RNA Pol II, and they usually undergo 5′- m7G capping, splicing, nonsense-mediated decay (NMD) and possess 3′-end poly(A) tails, thus resembling mRNA biogenesis [11,17]. A category of lncRNAs which do not obey these rules is known as circular RNAs (circRNAs), whose biological function remains largely unclear [17]. Notably, another group of lncRNAs is transcribed by Pol III, particularly those that functionally interact with the Pol II-dependent transcription machinery, possibly to uncouple the production of these ncRNAs from the Pol II transcriptional reaction that they regulate [22,23]. In addition, they exhibit no or minimal open reading frames (ORFs). In the latter case, micropeptides are produced from these restricted ORFs, which often have functional significance [8].

Due to the many functions that they perform and the variety of biological processes in which they are implicated, a classification of lncRNAs has been proposed according to the following criteria [10,17]:

*(i)*  *Genomic location of lncRNAs.* This classification involves: (a) intergenic lncRNAs (lincRNAs)—loci encoding such transcripts are located in the intervening regions among protein coding genes; (b) intronic lncRNAs—these transcripts are derived from introns that separate the coding exons of a gene.
*(ii)*  *Location context.* In this respect, lncRNAs relate to sense- or antisense-derived transcripts: (a) the former are transcribed from the sense strand and may overlap partly or completely with the entire sequence of a protein coding gene; (b) the antisense-derived transcripts originate from the antisense strand and can emerge from three routes: (1) antisense strand-derived transcripts may overlap with an exon of a sense gene, (2) transcripts may overlap with the intron of a sense gene, and (3) transcripts may cover the entire protein-coding sequence.
*(iii)*  *Effect exerted on DNA sequences*. Cis- and trans-lncRNAs: the former regulate the expression of genes in close genomic proximity, while the latter regulate the expression of distant genes.
*(iv)*  *Mechanism of function.* According to the function that they exert, lncRNAs are categorized into the following groups: (a) Transcriptional regulation. This group involves lncRNAs that are implicated in chromatin remodeling and transcriptional interference, including enhancer lncRNAs (eRNAs) that are transcribed from enhancers; (b) Post-transcriptional regulation. This group comprises lncRNAs that participate in splicing regulation and post-transcriptional regulation (mRNA stability); (c) Translational control, by facilitating or repressing this process; (d) Other functions, such as protein localization, telomere replication and RNA interference.
*(v)*  *Targeting mechanism*. This is a heterogeneous group of lncRNAs due to the multiple mechanisms of action and interactionsubstrates that they present or recognize, respectively. There are several subdivisions in this category: (a) signal dependency as lncRNAs show cell type-specific expression and response to diverse stimuli; (b) decoy role, since certain lncRNAs can bind and titrate away an RNA or protein target, without any further function(s); (c) guiding ability as they bind proteins and then direct the localization of the ribonucleoprotein complex to specific targets; (d) scaffolding capacity, since such lncRNAs serve as central platforms for the concurrent binding of multiple proteins to form ribonucleoprotein complexes. LncRNAs can also be grouped together based on the nature of the interactions that they establish with their targets: RNA–RNA pairing, RNA–DNA hybrids, RNA structure-mediated interactions and protein linkers.

## 3. The DNA Damage Response and Repair (*DDRR*) Network

DNA serves as a repository for genetic information; therefore, all living cells rely on a complex protein network, termed DDRR, to preserve its integrity [1,2]. Given the wide range of DNA lesions that are inflicted by numerous sources in the genetic material, the DDRR comprises several modules capable of recognizing and resolving specific types of those genetic lesions [1,2]. DDRR is hierarchically structured in a signaling cascade that sequentially involves: DNA damage sensors, signal mediators, signal transducers and, finally, effectors to recruit the appropriate DNA repair module and impose a global cellular response [1,2].

Two broad categories of genetic aberrations occur and accumulate in the genome, namely chemically induced changes at the nucleotide level and single- or double-strand breaks (*DSBs*) in the DNA helix [1,24]. Of all these types of DNA damage, the most deleterious ones are the DSBs, since, on one hand, they are deemed lethal if they are left unattended, while, on the other hand, if they are not properly repaired, they become a source of genomic instability, a hallmark of cancer and other diseases [1,24].

DSBs can arise from a variety of endogenous or exogenous sources, such as oncogene-induced replication stress, reactive species (*RS*), unrepaired mutations leading to stalled replication forks and ionizing radiation [1,4,25]. Genomic rearrangements such as deletions, insertions and translocations severely affect the genome’s integrity and are the consequences of defective DSB repair, often resulting in oncogene activation and tumor suppressor silencing [1,24,26]. In normal cells, DSBs are predominantly repaired by either the Non-Homologous DNA End Joining (NHEJ) or Homologous Recombination (HR) pathways [1], and the selective activation of DSB repair pathways depends on a plethora of factors, such as the complexity of the break [1,27,28].

In mammalian cells, while NHEJ is active throughout the cell cycle, its prominent action is observed during the G0 and G1 phases of the cell cycle [1,29,30,31]. Briefly, NHEJ is promoted by the signaling mediator 53BP1 and its first step involves binding of the Ku70-Ku80 heterodimers to each end of a DSB to facilitate the recruitment and assembly of the DNA–PK complex. In turn, this complex processes the DNA ends, along with the nuclease Artemis, and finally increases the recruitment of the XLF-XRCC4-DNA ligase IV complex, which carries out the DNA end rejoining reaction. The requirement to process the DNA ends in order to join them directly renders NHEJ an error-prone DNA repair mechanism [1].

On the other hand, HR occurs during the S and G2/M2 phases of the cell cycle and employs the sister chromatid as a template for repair [1]. The main molecular events in HR involve DSB recognition by the MRN complex (MRE11/RAD59/NBS) followed by DNA end resection by CtIP and EXO I, with the resulting single-stranded 3′ overhangs being RPA coated, thus facilitating RAD51 loading. Next, the RAD51 single-stranded DNA (ssDNA) nucleoprotein filament invades the homologous chromatid, forming a D-loop, and is extended along the recipient homologous DNA duplex by DNA polymerase (Pol δ). At this point, HR repair can follow two distinct routes. One is the double-strand break repair (DSBR) pathway, where, after strand invasion and synthesis, the other DSB end can be bound, leading to the generation of a two Holliday junction (HJ) intermediate. Following gap DNA synthesis and ligation, the intermediate structure is resolved at the HJs in a non-crossover or crossover manner. The other route is synthesis-dependent strand annealing (SDSA), during which the extended single-strand end is annealed to the ssDNA on the second complementary side of the DSB, followed by DNA gap-filling and ligation. The repair product from SDSA is always non-crossover. Both DSBR and SDSA are generally considered error-free mechanisms. Alternative HR repair modules involving recombination across regions with incomplete homology can also take place and are error-prone repair processes [1].

## 4. LncRNA and DSB Repair

**Figure 1:**
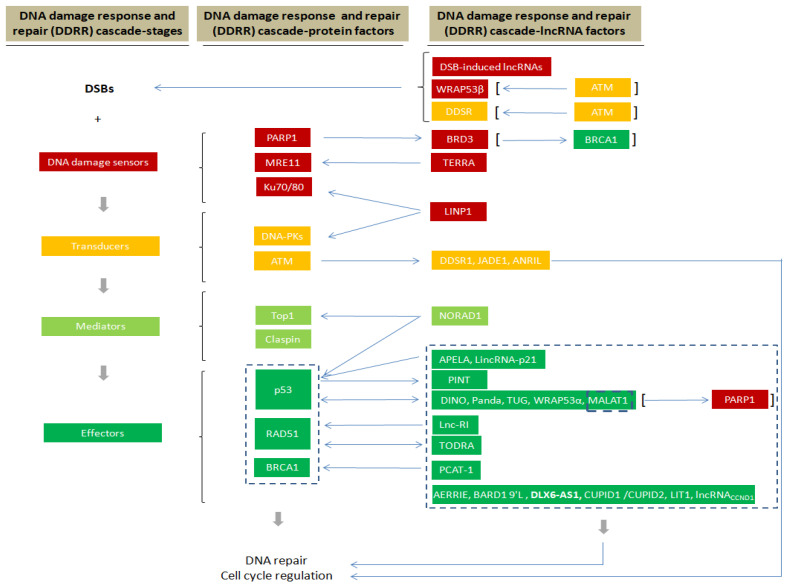
Schematic illustration of how lncRNAs are involved in the various stages of DDRR signaling.

**Figure 2:**
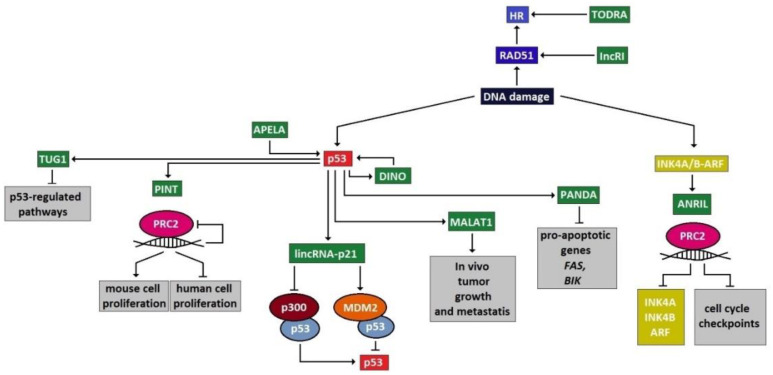
Schematic illustrating the different levels of DDRR regulation exerted by lncRNAs (presented in green color). LncRNAs interact with various components of the DDRR network, by either promoting or inhibiting their functions.

The majority of the DDRR network components that are predominantly investigated as functional factors have been identified as proteins. However, a growing body of evidence has uncovered an unexpected role for lncRNAs as versatile and vital components in this genome-preserving machinery [3]. Interestingly, the roles of these new players are not restricted to a specific function but are rather involved in several aspects of the DDRR activation, exerting a multilevel and even hierarchical control. As presented below, in response to DNA damage, and particularly upon DSB occurrence, lncRNAs are implicated in all DDRR signaling stages, namely DSB sensing, signal mediation and transduction and effector activity, either as targets of the protein components or as their regulators (Figure 1). As regulators of DDRR, lncRNAs can impact the expression of DDRR protein components, by exerting epigenetic, transcriptional, translational or protein stability control [17]. Other lncRNAs are implicated in facilitating the assembly and/or localization of DDRR-associated protein complexes, while certain lncRNAs serve to signal positions of DNA damage and even link DSB ends (Figure 2) [3].

### 4.1. LncRNAs Involved in DSB Sensing

LncRNAs can localize at DSBs either directly or through DDRR sensors. In the first case, an intriguing recent finding concerns the discovery of damage-induced transcription at sites of DNA breaks [3], involving non-canonical transcription at identified sites of DNA damage, and resulting in the production of mainly small non-coding RNAs (sncRNAs). These sncRNAs derive from the processing of lncRNAs and are named DNA damage response RNAs (DDRNAs). More specifically, DDRNAs are DROSHA- and DICER-regulated products, generated from the processing of damage-induced lncRNAs (dilncRNAs) that are transcribed by RNA Poll II at sites of DSBs. DilncRNAs have been proposed to function as scaffolds linking the DSB ends [3,32]. Moreover, they can adopt a double-stranded RNA conformation or generate RNA: DNA hybrids. In particular, assembly of the latter structure facilitates the loading of early HR repair factors at sites of DSBs. Nevertheless, if not properly resolved, these hybrids can be detrimental by preventing the recruitment of late HR factors, promoting mutagenesis [3,32].

Certain lncRNAs have been shown to facilitate the loading of either single components or assembled DDRR modules at DSBs to aid in repair execution. Notably, the “WD40 encoding RNA antisense to p53” (WRAP53β) lncRNA is a partial antisense to the TP53 gene, which has the ability to recruit and stabilize RNF8 at DSBs [33]. Additionally, the ATM target “DNA damage-sensitive RNA1” (DDSR1) lincRNA has been proposed to complex with the heterogenous nuclear ribonucleoprotein-U-like 1 (hnRNPUL1), to mediate the recruitment of BRCA1-RAP80 at the sites of DSBs [34]. Of note, DDSR1 has also been found to play a role in DNA end resection during HR repair [35], thus exhibiting a dual level of control, as a sensor and effector, within the DDRR cascade.

The p53-upregulated “Metastasis-associated Lung Adenocarcinoma Transcript 1” (MALAT1) lncRNA [36,37] is an approximately 7 Kb lincRNA that is evolutionarily conserved and has been found to be associated with several malignancies [38]. MALAT1 has been reported to form trimeric complexes with PARP1 and LIG3 in vivo, possibly being involved in the regulation of the Alt-NHEJ pathway for DSB repair [39]. PARP1 also recruits BGL3 lncRNA at DSBs to facilitate the upload of the BARD1/BRCA1 complex at these sites [40].

Finally, “Telomeric Repeat-containing RNA” (TERRA) is a lncRNA with a dual role. On one hand, it interacts with LSD1 to regulate meiotic recombination 11 (MRE11) activity at uncapped telomeres [41]. On the other hand, TERRA lncRNAs have been observed to generate DNA–RNA hybrids at telomeres, which can enhance HR between telomeres [42]. Moreover, the “LncRNA In Non-homologous end joining Pathway” (LINP1) exerts also a dual function as a scaffold, in order to facilitate: (i) the interaction of Ku70-Ku80 and DNA-PKcs and (ii) the formation of complexes with chromatin [43].

### 4.2. LncRNAs Acting at Transducer/Mediator Level

ATM activation leads to the induction of the ANRIL lncRNA (antisense noncoding RNA from the *INK4A* locus) [44]. The previously mentioned DDSR1 lincRNA is also an ATM target that is involved in the HR repair pathway, as its inactivation results in sensitization to PARP1 inhibitor administration (Figure 1) [45].

“LncRNA-Jade family plant homeodomain (PHD) finger” (lncRNA-JADE) is another ATM-induced target in response to DNA damage. Specifically, lncRNA-JADE upregulates JADE1, a key factor of the HBO1 histone acetylation complex, thus triggering histone H4 acetylation during DDRR [46]. It has been postulated that lncRNA-JADE also interacts with BRCA1 and is, therefore, recruited to the p300/CBP complex. LncRNA-JADE has been found aberrantly overexpressed in human breast tumors [46].

The lncRNA “Non-coding RNA Activated by DNA damage” (NORAD) was found to interact with RNA Binding Motif Protein X-Linked (RBMX) and control its ability to assemble a ribonucleoprotein complex that includes critical genome instability suppressors such as topoisomerase I (TOP1), ALYREF and the PRPF19–CDC5L complex [47]. Silencing of NORAD or RBMX resulted in increased frequency of chromosome segregation defects, reduced replication-fork speed and altered cell-cycle progression.

### 4.3. LncRNAs Acting at the Effector Level

Multiple lncRNAs have an established role as effectors in the DDRR signaling network. Of particular interest are those targeted by p53. PINT is a lincRNA that acts as a transcriptional target of p53 and a positive regulator of cell proliferation and survival in mouse cells, while it has been shown to function as a negative regulator in human cells [48,49]. In both humans and mice, PINT represses PRC2 by targeting specific gene loci, thereby affecting cell proliferation, but with apparently different outcomes between the two species [48,49]. Another lncRNA that is activated by p53 is TUG1, which also acts on chromatin to repress p53-regulated pathways [50,51]. The previously mentioned ANRIL lncRNA is encoded by the INK4A/B-ARF locus on chromosome 9p21, known to frequently undergo copy number alteration in many tumors [44]. ANRIL also recruits the PRC complex to repress the transcription of the tumor suppressor genes INK4A, INK4B and ARF and indirectly affects HR by altering cell-cycle checkpoints [44].

MALAT1 is also upregulated by p53 in response to DNA damage, similarly to TUG1. Apart from its role in damage sensing, numerous other proteins that are involved in transcription, RNA processing, translation, protein degradation and metabolism have been found among its various partners [52]. Moreover, MALAT1 has the ability to sequester DBC1, a partner of the SIRT1 deacetylase that regulates p53 deacetylation [52].

Another DNA damage-induced lncRNA named “P21 Associated NcRNA DNA damage Activated” (PANDA) is also expressed in a p53-dependent manner and represents an antisense-transcribed lncRNA of approximately 5 Kb that is located upstream of CDKN1A [53]. PANDA functions as a decoy for the subunit alpha (NF-YA) to repress the pro-apoptotic genes FAS and BIK. Additional lncRNAs that exert control at the transcriptional level involve the lincRNA-p21, which is located 15 Kb upstream of the CDKN1A locus and functions as a repressor of the p53 transcriptional program [53,54]. LincRNA-p21 is a 3.1-Kb-long RNA with its own promoter and is antisense-transcribed. Its mode of action involves interaction with the heterogenous nuclear ribonucleoprotein-K (hnRNP-K), a component of repressor complexes targeting p53-regulated genes. LincRNA-p21 has been found to interact with the E3 ligase MDM2, leading to stabilization of the MDM2–p53 interaction, resulting in p53 degradation. Additionally, lincRNA-p21 downregulates the p300–p53 interaction, which results in p53 activation [53,54]. In contrast, the lncRNA APELA interacts with heterogenous nuclear ribonucleoprotein-L (hnRNP-L), preventing the complexing of p53 with hnRNP-L and facilitating, in turn, p53-mediated apoptosis.

The WRAP53 locus encodes for three isoforms (α, β, γ), of which WRAP53α has been demonstrated to interact with p53 mRNA, affecting, in turn, p53 protein levels [55]. Another level of interactions concerns protein stabilization. A recently identified lncRNA termed “Damage Induced Noncoding” (DINO) is a 951 nt RNA that is induced by p53 and also located upstream of CDKN1A [56]. Notably, DINO interacts with the p53 protein, stabilizing it and, thus, promoting its transactivation capability. “Prostate Cancer Associate Transcript 1” (PCAT-1) is a prostate cancer-specific lincRNA involved in DSB repair [57]. Its increased levels suppress BRCA2 and therefore impair the HR pathway [58], representing a typical “BRCA-ness” example, beyond gene mutation. It has been proposed as a predictive response biomarker for treatment with PARP1 inhibitors. PCAT-1 is predominantly cytoplasmic and downregulates BRCA2 mRNA in a post-transcriptional manner.

Expression of the “Transcribed in the Opposite Direction of RAD51” (TODRA) lncRNA is controlled by the same regulatory region that induces RAD51, but is transcribed reciprocally to RAD51, in an E2F1-dependent manner [59]. TODRA cooperates with RAD51 in HR-mediated DSB repair. Its expression is deregulated in human malignancies, leading to genomic instability and tumor progression [39].

Another lncRNA that stabilizes RAD51 is the “long noncoding RNA Radiation Induced” (lnc-RI) [43]. Lnc-RI functions as a “sponge” towards miR-193a-3p, preventing its suppressive activity against RAD51. Consequently, it promotes RAD51-mediated HR repair. Nevertheless, in another report, lnc-RI was found to affect colorectal cancer (CRC) cell growth and radiosensitivity by upregulating the NHEJ repair pathway through LIG4 stabilization [60].

“BRCA1-associated RING Domain protein 1 9′L” (BARD1 9′L) is transcribed from an alternative intronic promoter of the BARD1 gene and upregulates the expression of an oncogenic BARD1 isoform. Normally, BARD1 stabilizes the BRCA1 protein, thus facilitating HR repair. By promoting the expression of an oncogenic isoform, BARD1 9′L interferes with this process in certain malignancies and is accompanied by reduced RAD51 foci generation and decreased nuclear accumulation of the BRCA1 protein [61,62].

Two estrogen-regulated long noncoding RNAs, “CCND1-UPstream Intergenic DNA repair 1 and 2” (CUPID1 and CUPID2), are expressed from a bidirectional promoter from an intergenic region at 11q13 [63]. CUPID1 and CUPID2 are predominantly expressed in hormone-receptor-positive breast tumors and are implicated in modulating pathway choice (HR versus NHEJ) for the repair of DSBs. Interactions of lncRNAs with components of the DDRR are depicted in Figure 2.

“DLX6 Antisense RNA 1” (DLX6-AS1 or Evf 2) is a lncRNA that interacts with Brahma-related gene-1 (BRG1) and DLX1. It has an inhibitory role on RNA-dependent chromatin remodeling [42]. Notably, BRG1 functions as the catalytic ATPase of the SWItch/Sucrose Non-Fermentable (SWI/SNF) complex that is phosphorylated by ATM and participates in DSB repair [64].

The recently discovered AERRIE lncRNA was shown to be expressed preferentially in endothelial cells and facilitate DDRR [65]. Specifically, AERRIE interacts with Y-Box Binding Protein 1 (YBX1) to form a complex in order to facilitate DNA damage signaling and repair.

A group of lncRNAs termed lncRNA_CCND1_ are expressed in response to ionizing radiation from the Cyclin D1 (CCND1) promoter locus [34,35]. lncRNA_CCND1_ subsequently interact with the RNA-binding protein “Translocated in Liposarcoma” (TLS) to suppress CCND1 transcription through inhibiting the histone acetyltransferase complex CBP/p300, resulting in cell proliferation cessation [34,35].

Expression of the CDKI p57^KIP1^ has been shown to be under epigenetic regulation in mammals through an imprinted control region (ICR) [66]. Specifically, in normal cells, p57^KIP1^ is expressed from the maternal allele, as the paternal one is suppressed by a lncRNA termed “Long QT Intronic Transcript 1” (LIT1) [66]. LIT1 lncRNA is transcribed from an intron of the paternal KCNQ1/KvDMR-ICR locus on the 11p15.5 chromosome arm and downregulates several adjacent genes, including p57^KIP1^, in the same chromosomal arm [66]. We have previously observed that, in a subset of patients with non-small cell lung cancer, LIT1 lncRNA was abnormally overexpressed and inversely correlated with p57^KIP1^ expression levels, suggesting that LIT1 lncRNA could block p57^KIP1^-dependent cell-cycle checkpoint [67].

## 5. Significance of lncRNAs in DDRR

The initial observations that a large proportion of the eukaryotic genome is transcribed but, eventually, only a small fraction of the generated RNAs are translated into protein “players” seemed intriguing at the time [17]. Why would cells invest so much energy in producing untranslatable RNA, or, in other words, what is the functional advantage gained by this strategy?

At first, ncRNA was considered to be “dark matter”, but evidence progressively came to light unveiling an increasing range of cellular functions that rely on the presence of ncRNAs for normal execution [3,5,9]. Nowadays, due to the multiplicity of the roles that they perform, non-coding RNAs—including lncRNAs—are collectively identified as “smart” molecules, involved in various aspects of normal physiology [3,17]. Furthermore, deregulated expression of ncRNAs has been documented as a primary source for disease initiation and/or progression [17].

Of particular importance are those lncRNAs involved in the DDRR network due to its vital role in preserving the integrity of the eukaryotic genome. Taking into account the rapidly increasing numbers of discovered and/or functionally characterized lncRNAs against the protein components of the DDRR network, it is tempting to speculate that lncRNAs engaged in the DDRR may, in fact, outnumber the protein factors participating in it. In any case, the intriguing question of why RNA transcripts are deployed in this critical network still remains unanswered. To address the role of lncRNAs in cellular physiology, several cell systems have been implemented, such as stem cells or induced pluripotent stem cells (iPSC), uncovering their dynamic role in cell fate determination [68]. To this end, CRISPR-mediated radiation modifier screens have identified lncRNA therapeutic targets in gliomas using hPSC-derived brain organoids [69]. Patient-derived organoids which are capable of faithfully recapitulating the tissue of origin [70,71,72] have been also employed to study the role of lncRNAs in cancer [73].

A plausible hypothesis regarding lncRNA evolution could envisage that, since RNA once served as an ancestral life-coding molecule [74], ncRNAs may be remnants that have been selected and preserved, as they may offer survival or fitness advantages. This likely applies in the case of the DDRR network, as the DDRR circuitry is constantly engaged in recognizing a vast array of DNA damage types, activation of the appropriate response checkpoints and setting in motion a plethora of metabolic cellular processes that range from DNA repair to halting cell proliferation, activation of senescence or driving various types of cell death [1]. These stress-derived processes entail differential energy demands and require timely responses [1]. Notably, at the pathological level, stress conditions are characterized by the cytoplasmic accumulation of stress granules, representing dense cytosolic aggregations lacking a membrane, which, apart from proteins, are also composed of RNAs [1]. Under stress conditions, critical protein factors are often exhausted, generating bottlenecks in the DDRR flow, leading to accumulations of genetic aberrations, infliction of genomic instability and the emergence of pathological phenotypes [1,75]. Thus, cells have possibly adapted to employ protein factors for critical hubs in the DDRR signaling and engage low-cost/rapidly produced supportive DDRR components via lncRNAs. Overall, the above observations highlight that the central dogma of biology may have additional ramifications, increasing the complexity of regulatory and functional networks within a cell [3].

## 6. Conclusions and Future Perspectives

DDRR impacts multiple cellular functions; however, addressing how such complex programs are orchestrated still remains a challenge [1,76]. The involvement of lncRNAs increases the complexity of this network. Therefore, an important issue is how lncRNAs shape and fine-tune the action of DDRR. Moreover, harnessing DDRR knowledge can offer windows of opportunity for treating diseases [1,76], particularly through exploitation of lncRNAs.

Within this context, the advent of high-throughput technologies has already generated large numbers of lncRNA entries in repository databases, many of which are still awaiting functional characterization. Nevertheless, the already characterized lncRNAs have signified their vital contribution to cellular physiology and the impact that their deregulation has on disease onset [17,73]. As such, there is an increasing interest in exploiting these molecules as disease biomarkers [17], as well as new targets in therapeutic approaches for disease treatment [73].
